# Tunnelling nanotubes between neuronal and microglial cells allow bi-directional transfer of *α*-Synuclein and mitochondria

**DOI:** 10.1038/s41419-023-05835-8

**Published:** 2023-05-18

**Authors:** Ranabir Chakraborty, Takashi Nonaka, Masato Hasegawa, Chiara Zurzolo

**Affiliations:** 1 Institut Pasteur, Université Paris Cité, CNRS UMR 3691, Membrane Traffic and Pathogenesis, Paris, France; 2 Université Paris Saclay, Gif-sur-Yvette, Paris, France; 3 Dementia Research Project, Tokyo Metropolitan Institute of Medical Science, Tokyo, Japan; 4 Department of Molecular Medicine and Medical Biotechnology, University of Naples Federico II, Naples, Italy

**Keywords:** Cell biology, Microglia, Cellular neuroscience

## Abstract

Tunnelling Nanotubes (TNTs) facilitate contact-mediated intercellular communication over long distances. Material transfer via TNTs can range from ions and intracellular organelles to protein aggregates and pathogens. Prion-like toxic protein aggregates accumulating in several neurodegenerative pathologies, such as Alzheimer’s, Parkinson’s, and Huntington’s diseases, have been shown to spread via TNTs not only between neurons, but also between neurons-astrocytes, and neurons-pericytes, indicating the importance of TNTs in mediating neuron–glia interactions. TNT-like structures were also reported between microglia, however, their roles in neuron-microglia interaction remain elusive. In this work, we quantitatively characterise microglial TNTs and their cytoskeletal composition, and demonstrate that TNTs form between human neuronal and microglial cells. We show that *α*-Synuclein (*α*-Syn) aggregates increase the global TNT-mediated connectivity between cells, along with the number of TNT connections per cell pair. Homotypic TNTs formed between microglial cells, and heterotypic TNTs between neuronal and microglial cells are furthermore shown to be functional, allowing movement of both *α*-Syn and mitochondria. Quantitative analysis shows that *α*-Syn aggregates are transferred predominantly from neuronal to microglial cells, possibly as a mechanism to relieve the burden of accumulated aggregates. By contrast, microglia transfer mitochondria preferably to *α*-Syn burdened neuronal cells over the healthy ones, likely as a potential rescue mechanism. Besides describing novel TNT-mediated communication between neuronal and microglial cells, this work allows us to better understand the cellular mechanisms of spreading neurodegenerative diseases, shedding light on the role of microglia.

## Introduction

Accumulation of neurotoxic protein aggregates in neurodegenerative diseases (NDs) such as Alzheimer’s disease (AD), Parkinson’s disease (PD), Huntington’s disease (HD), eventually lead to the death of burdened neurons that are unable to dilute the aggregates because of their post-mitotic nature, as well as because of the impairment of the degradative pathways [1, 2]. Although cells have different quality control mechanisms in place to clear misfolded proteins, such as the ubiquitin-proteasome system (UPS), and autophagy, continuous generation of aggregates renders these processes ineffective in maintaining proteostasis [2, 3]. Besides the role of intrinsic protein accumulation, neuronal death in NDs is also regulated in a non-cell autonomous manner by glial cells. Both astrocytes, the major glial cell type, and microglia, the tissue-resident macrophages of the brain, respond to neurodegenerating conditions by altering their cellular properties from a homoeostatic to a reactive phenotype, a phenomenon characteristic of neuroinflammation [4]. If extended beyond its initial stages of brain repair and neuroprotection, neuroinflammation can result in progression of neurodegeneration [5].

Alongside astrocytes, microglia are major responders to impaired neuronal proteostasis. Presence of protein aggregates drives microglia towards a reactive phenotype, that subsequently initiates various direct and indirect routes leading to neuronal death [6]. In cases of PD, degeneration of dopaminergic neurons begins at the substantia nigra pars compacta (SNpc). In later stages, the pathology spreads and *α*-Syn deposition has been observed in different other regions of the brain, implicating the propagative nature of these aggregates in a “prion-like” mechanism [7–9]. A mechanism of disease propagation is via the release of *α*-Syn aggregates by neurons, which eventually binds to, and is engulfed by, surrounding neurons [10–13]. Besides neurons, neighbouring astrocytes and microglia express cell-surface receptors that recognise *α*-Syn as a ligand, such as toll-like receptor 2 (TLR2), Fyn kinase and scavenger receptor CD36, and FcγRIIB, that drives an NLRP3 inflammasome-driven proinflammatory signalling cascade [14–18]. Consequently, microglia in different regions eventually acquire a reactive phenotype. Furthermore, upon binding of neuron-released *α*-Syn to TLR4, microglia have been reported to upregulate the expression of p62/SQSTM in an NF*κ*B-dependent manner that eventually leads to the autophagic clearance of the aggregates [19].

In addition to secretion-based mechanisms, recent in vitro and ex vivo evidence support the involvement of tunnelling nanotubes (TNTs) in spreading of neurodegenerative pathologies. First described in 2004, TNTs are thin, membrane-enclosed, F-Actin-rich protrusions that connect cells over long distances [20]. Functionally, TNTs are able to transfer cargoes of different kinds between the connected cells, like Ca^2+^ signals, messenger- and micro-RNAs, organelles such as lysosomes and mitochondria, pathogens, apoptotic signals, and ND-protein aggregates [21, 22]. Amyloidogenic proteins such as prions [23], amyloid *β* [24], tau [25], mHTT [26], and *α*-Syn [27] have been reported to use TNTs as a route for spreading from one cell to another in in vitro co-cultures and in brain slices. Interestingly, *α*-Syn aggregates closely associate with lysosomes to move to an uninfected neuronal cell and initiate aggregate seeding [28]. *α*-Syn fibrils can also propagate between neurons and astrocytes [29, 30], as well as neurons and pericytes [31] via TNTs. Recently, an elegant work using mouse primary microglia, human monocyte-derived microglia-like cells, and post-mortem human brain samples, provided qualitative data showing the formation of TNTs allowing the spreading of *α*-Syn aggregates between microglial cells and leading to their eventual degradation [32]. However, to understand the role of microglia in the progression of NDs, it is of capital importance to investigate whether TNTs form between neurons and microglia and to what extent do these aggregates move between these cells while using TNTs to do so.

Using both quantitative and qualitative live imaging approaches, we report here for the first time the presence of functional TNTs between neuronal and microglial cell lines that allow movement of mitochondria and *α*-Syn aggregates between these cells both uni- and bi-directionally. We quantitatively assess the extent of such transfers, revealing a bias of *α*-Syn transfer from neuronal cells to microglia, and mitochondrial movement from microglia preferably to unhealthy (*α*-Syn-containing) neuronal cells. Our in vitro results point towards TNTs as a possible major route of neuron-microglia interactions in normal and neurodegenerating contexts and support a rescue function for microglia, thereby advancing our understanding neuron-glia interactions.

## Materials and methods

### Cell culture and *α*-Syn treatment

Human neuroblastoma cell line SH-SY5Y (hereafter referred to as neuronal cells) were grown in undifferentiating conditions in RPMI1640 media (Euroclone, ECB2000L), supplemented with 10% FCS (Eurobio Scientific, CVFSVF00-01) and 1% Penicillin-Streptomycin (Pen-Strep, Gibco, 15140-122) as described before [33]. Human microglial clone 3 (HMC3) cell line [34], a kind gift from Dr Aleksandra Deczkowska, Institut Pasteur, was grown in DMEM media (Sigma-Aldrich, D6429), supplemented with 10% FCS and 1% Penicillin-Streptomycin. Cells were maintained in a humidified incubator at 37 °C, and passaged at 80–90% confluency. Cells were seeded at 1:5 ratio for maintenance in culture, and counted before seeding for particular experiments.

Preformed fibrils were prepared as described previously [35]. Briefly, human WT *α*-Syn in pRK172 vector was transformed into *Escherichia coli* BL21 (DE3), followed by purification with RP-HPLC. The obtained *α*-Syn fibrils were then conjugated with AlexaFluor 488 or 568 (Invitrogen) using manufacturer’s protein labelling kit. Fibrils for experiments were prepared as previously described [28]. For treatment with *α*-Syn, cells were seeded in uncoated 6-well plates (Falcon, 353046) at a density of 400,000 cells/well, and grown for 24 hours (h). Briefly, fluorophore-conjugated fibrils were diluted in growth medium (500 nM) without the addition of any intracellular delivery agents and sonicated (BioBlock Scientific, Vibra Cell 75041) for 5 min (80% amplitude, pulse- on: 5 s, off: 2 s). Sonicated fibrils were then added to the cells for 16 h of incubation. After 16 h, cells were washed three times with 1:3 trypsin solution diluted in 1X PBS, and processed further for subsequent experiments.

### Labelling TNTs

Owing to the fragile nature of TNTs, cells were handled with utmost care to minimize physical stress. After cells reached sub-confluency (~70%), a two-step fixation protocol was used to preserve TNTs as described previously [36]. Briefly, cells were fixed initially with a fixative containing glutaraldehyde (GA) (0.05% GA, 2% paraformaldehyde (PFA), 0.2 M HEPES buffer in 1X PBS) for 15 minutes at room temperature (RT), followed by a second fixation without GA for 15 minutes at RT (4% PFA, 0.2 M HEPES buffer in 1X PBS). Following this, cells were washed three times with 1X PBS, and stained for plasma membrane with 3.33 *μ*g/mL wheat germ agglutinin (WGA, Life Technologies, Thermo Fisher Scientific WGA488- #W6748, WGA647- #W32466), and/or F-Actin with Phalloidin (1:250 v/v, diluted in 1X PBS, Invitrogen, Thermo Fisher Scientific, A12380) for 15 minutes at RT in dark. In co-culture of WT SH-SY5Y neuronal and HMC3 microglial cells (Fig. 6D), microglial cells were loaded with Cell Tracker Red CMTPX (Invitrogen, C34552) at a working concentration of 1 *μ*M for 30 minutes at 37 °C.

### Immunocytochemistry

Cells were grown to sub-confluency and rinsed once with freshly-prepared and warmed cytoskeleton buffer (60 mM PIPES buffer, pH 6.9; 25 mM HEPES, 10 mM EGTA and 2 mM MgCl_2_ in milli-Q water). Cells were then fixed with 0.05% GA and 4% PFA diluted in cytoskeleton buffer for 20 minutes at 37 °C. PFA was quenched with 50 mM NH_4_Cl for 15 minutes at RT, followed by three washes with 1X PBS. Cells were then permeabilized with 0.1% Triton X-100 in 1X PBS (0.1% PBSTx) for 3 min, followed by blocking with 2% bovine serum albumin (BSA, Sigma Aldrich, A9647). Primary antibody against *α*-Tubulin was diluted (1:500, Sigma Aldrich, T9026) in 2% BSA and cells were incubated overnight at 4 °C. The next day, cells were washed three times with 0.1% PBSTx, and incubated with AlexaFluor488-conjugated secondary antibody diluted in 2% BSA (1:500, Invitrogen, Thermo Fisher Scientific, A11029) against the primary antibody for 40 minutes at RT. Cells were then washed three times with 0.1% PBSTx and incubated with AlexaFluor568-conjugated Phalloidin (1:250 v/v, diluted in 1X PBS) for 15 minutes at RT in dark. Cells were washed three times with 1X PBS, incubated with AlexaFluor647-conjugated WGA (3.33 *μ*g/mL diluted in 1X PBS) for 15 minutes at RT, washed three times with 1X PBS before mounting with Aqua-Poly/Mount (Polysciences Inc., 18606-20).

### Transfer assay of *α*-Syn

To assess for the movement of *α*-Syn between neuronal and microglial cells, a co-culture strategy was adopted. Assessing for transfer from neuronal cells to microglia (N → M), SH-SY5Y cells were loaded with *α*-Syn fibrils (donor cells) and co-cultured in a 1:1 ratio with HMC3 (acceptor cells) on coverslips in 24-well plates. In another condition to check for transfer in the opposite direction, microglia were the donor cells while neuronal cells were acceptors (M → N). Cells were grown for 24 h, fixed to preserve TNTs (as mentioned previously), stained for membrane, and mounted on slides. Cells were imaged and the number of *α*-Syn-positive acceptor cells were counted manually along different z-planes of optically-sliced images to ensure that the *α*-Syn particles were inside the cell. Additionally, the number of *α*-Syn particles per acceptor cell were also counted.

To negate the possibility of transfer in a secretion-dependent manner, donor and acceptor cells were grown in mono-cultures in different dishes. Conditioned medium from donor cells was added to acceptor cells (on coverslips in 24-well plate) for 24 h, followed by fixation and mounting. Cells were imaged and the number of *α*-Syn puncta per acceptor cells were counted. Data from co-culture condition was normalised to secretion control by subtracting the average of secretion control group.

### Transfer assay of mitochondria

Transfer of mitochondria was assessed from microglia to neuronal cells with or without *α*-Syn (referred as M → N ( + *α*-Syn) and M → N (WT) respectively). Microglial mitochondria were labelled with MitoTracker Red CMXRos (500 nM, Invitrogen, Thermo Fisher Scientific, M7512) diluted in growth medium for 30 min at 37 °C before seeding for co-culture. Neuronal cells (WT or + *α*-Syn; acceptor cells) were co-cultured in a 1:1 ratio with microglia (donor cells) and grown for 24 h before fixation as mentioned previously. Cells were imaged and the number of MitoTracker red-positive acceptor cells were counted manually along different z-planes of optically-sliced images to ensure that the particles counted were inside the cell. Additionally, the number of mitochondrial particles per acceptor cells were also counted.

For secretion control, conditioned medium from mitochondria-labelled microglia grown in mono-culture was added to acceptor cells (WT and + *α*-Syn, grown on different coverslips in 24-well plates) for 24 h before fixation and mounting. Data obtained from co-culture group was normalised for secretion control as mentioned above for *α*-Syn transfer assay.

### Fixed-cell imaging

Images were acquired using Zeiss LSM900 confocal microscope, 40X/1.3 NA oil objective and 0.8x zoom. All images were acquired with the “confocal” setting for optimal pixel sampling of the regions of interests. All the images were acquired at 16 bits, without any averaging across pixels. Optical slices were designated at a constant interval of 0.45 *μ*m, from the bottom of the cell to the top. Depending on the spread of cells on coverslips, the number of optical sections acquired ranged between 15 and 30. 3-D rendering of confocal images were done using Imaris (9.9.1). Snapshots of 3-D view are represented in Figs. 4E and 5G-H.

### Live-cell imaging

For live cell imaging, cells were grown in 35 mm glass-bottom dishes (Ibidi microdish, 81156, No. 15 coverslip bottom- thickness 0.17 mm) for 24 h. Cells were loaded with CellMask Deep Red Actin tracking stain (Invitrogen, A57245) (diluted in growth media to a working concentration of 1X from a 1000X stock solution) for 30 minutes at 37 °C. Cells were washed three times with growth media before imaging. Time-lapse images were acquired using Nikon Eclipse Ti2 spinning disk microscope, 60X/1.4 NA oil objective every minute for 30 minutes (30 frames). Optical sections were set at optimum for acquisition.

### Image analysis and statistics

All time-fixed and time-lapse images were analysed using FIJI image processing software. For analysing the proportion of TNT-connected cells, images were analysed using ICY software (https://icy.bioimageanalysis.org/). Images were assessed for TNT connections along the optical slices, and cells were marked to be connected if there existed one or more TNT(s) in the middle stacks connecting two cells. From a field of view, the number of connected cells were counted and normalised to the total number of cells in the region to represent the proportion of TNT-connected cells. Heterotypic TNTs were counted and represented in proportion using the following equation:

1 $$\frac{{(\frac{{Number\,of\,SH - SY5Y\,connected\,heterotypically}}{{Total\,number\,of\,SH - SY5Y\,cells\,connected}}) + (\frac{{Number\,of\,HMC3\,connected\,heterotypically}}{{Total\,number\,of\,HMC3\,cells\,connected}})}}{{(Total\,number\,of\,SH - SY5Y\,cells\,connected + Total\,number\,of\,HMC3\,cells\,connected)}} \times 100$$

Using this equation, the number of SH-SY5Y neuronal cells and HMC3 microglia connected to each other by TNTs were normalised to the total number of connected cells.

Lengths of TNTs were measured using membrane fluorescence, from one end of the protrusion to the end towards the opposing cell. Diameter of TNTs were measured by drawing a line perpendicular to the protrusion, and measuring the full-width half-maxima (FWHM) of the gaussian distribution of intensity of membrane fluorescence.

Movies were created at a rate of 2 frames per second. All experiments were done in triplicates, with the number of cells analysed in each group and experiments mentioned in the figure legends. No a priori power analysis was done to calculate the sample size. Graphs were plotted in GraphPad Prism 7.0, and appropriate statistical tests were implemented, as mentioned in the figure legends. Statistical significance was calculated with an *α*-value of 0.05. To determine the effect sizes and calculate Cohen’s d, Bland-Altmann plots were plotted with the estimation statistics online tool (www.estimationstats.com; [37]). The experimenter was not blind to the analyses performed.

## Results

### Tunnelling nanotubes connect HMC3 microglia

**Fig. 1:**
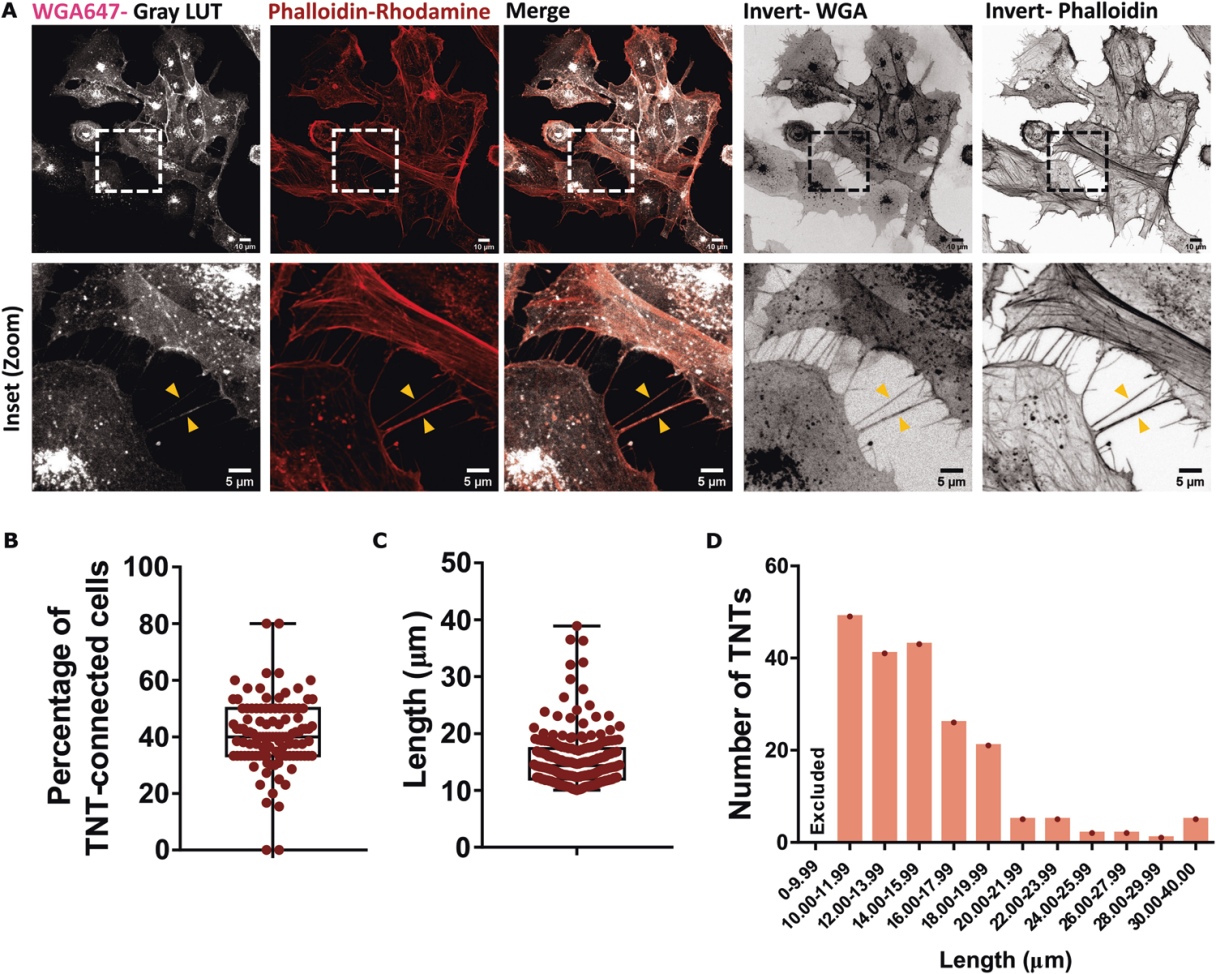
Tunnelling nanotubes are present between microglia. **A** HMC3 microglia stained for membrane with wheat germ agglutinin (WGA, grey) and F-Actin with phalloidin (red). Bottom panel represents the zoomed region depicted with boxes in the upper panel. Yellow arrowheads point towards TNTs. **B** Proportion of TNT-connected cells under normal growing conditions (*N* = 3 independent experiments, *n* = 111 regions of interests). **C** Lengths of the TNTs observed. **D** Distribution of TNTs based on their lengths. Structures less than 10 *μ*m were excluded from our analyses. *N* = 3 independent experiments, *n* = 200 TNTs for **C**, **D**.

A recent report provides evidence of TNT-like structures connecting murine primary microglia and human monocyte-derived microglia like cells [32]. Because no specific biomarker exists for these structures, the categorisation of cellular protrusions as TNTs requires several stringent criteria to be considered [36, 38]. Accordingly, our morphological identification of TNTs refers to membranous intercellular connections with a length of 10 μm and more (to differentiate them from filopodia) [39, 40], that hover above the substratum (in the middle stacks of an optically-sliced image) [41], and contain F-Actin. Using HMC3 microglial cell line, well-characterised for its antigenic profile and functional similarities with primary microglia [34], stained for membrane and F-Actin (Fig. 1A), we show that under normal growing conditions, 39.82% of HMC3 cells were connected by TNTs (Fig. 1B), with majority of their lengths being in the range of 10-20 μm (Fig. 1C, D) supporting the qualitative evidence of the existence of TNTs between microglia both in vitro, and in post-mortem human brain samples [32].

### Microglial TNTs contain F-Actin alone, or together with *α*-Tubulin

**Fig. 2:**
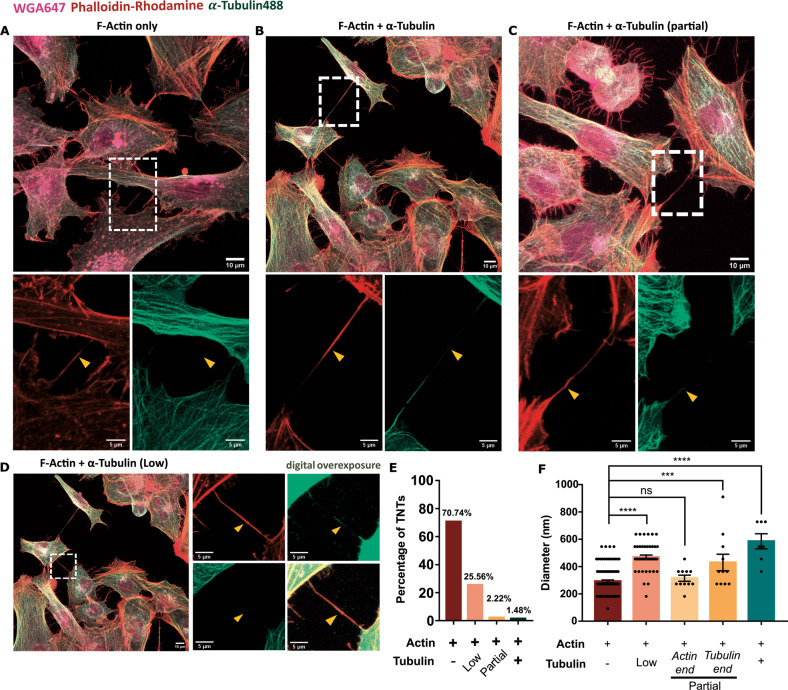
Cytoskeletal composition of microglial TNTs. **A**–**D** Immunostaining for *α*-Tubulin (green) and subsequent staining for F-Actin with phalloidin rhodamine (red) and membrane with WGA647 (magenta). **A** TNT containing only F-Actin. **B** TNT containing both F-Actin and *α*-Tubulin. **C** TNT partially containing *α*-Tubulin. **D** TNT containing low level of *α*-Tubulin. Yellow arrowheads in (A-D) point towards the TNT. **E** Proportion of TNTs with diverse cytoskeletal compositions. *N* = 3 independent experiments, *n* = 270 TNTs. **F** Diameter of TNTs with different cytoskeletal compositions. *N* = 3 independent experiments, *n* = 173 TNTs analysed; One-Way ANOVA with Tukey’s *post-hoc*; ns: non-significant, ***p < 0.001, ****p < 0.0001. Error bars represent mean ± SEM.

Although ‘thin’ or ‘canonical’ TNTs have been described to be rich in F-Actin filaments for a variety of cell types such as SH-SY5Y neuroblastoma cells [33], the presence of microtubule along with F-Actin have been reported in ‘thick’ TNTs, characteristic of cells of myeloid origin (‘thin’ TNTs: diameter <700 nm; and ‘thick’ TNTs: diameter >700 nm) [42, 43]. Of interest, quantitative assessment of the cytoskeletal composition of TNTs showed that differently from myeloid cells, most (70.74%) of the microglia TNTs contained only F-Actin (Fig. 2A, E). However, 29.26% of the TNTs contained microtubule along with F-Actin, albeit to different extents. In 25.56% of the TNTs, microtubule presence was detected at a very low level, visualised evidently only upon digital overexposure (Fig. 2D, E). In 2.22% of the cases, TNTs contained microtubule only up to a segment of the structure (Fig. 2C, E), whereas 1.48% of the TNTs contained microtubule throughout the length (Fig. 2B, E). As the presence of Tubulin has been reported to increase the thickness of TNTs, we next analysed the diameter of these structures, and observed a mean diameter of ~292 nm for only F-Actin containing TNTs, and ~585 nm for TNTs containing both F-Actin and Tubulin. In cases of TNTs containing Tubulin only to a partial length, the diameter was higher (~430 nm) towards the Tubulin-containing end as compared to only F-Actin-containing end of the TNT (~314 nm). TNTs with low levels of Tubulin also had an higher average diameter of ~470 nm (Fig. 2F). Taken together, these results suggest that microglia can be connected by both ‘thin’ and ‘thick’ TNTs.

### TNTs formed by microglia contain mitochondria

**Fig. 3:**
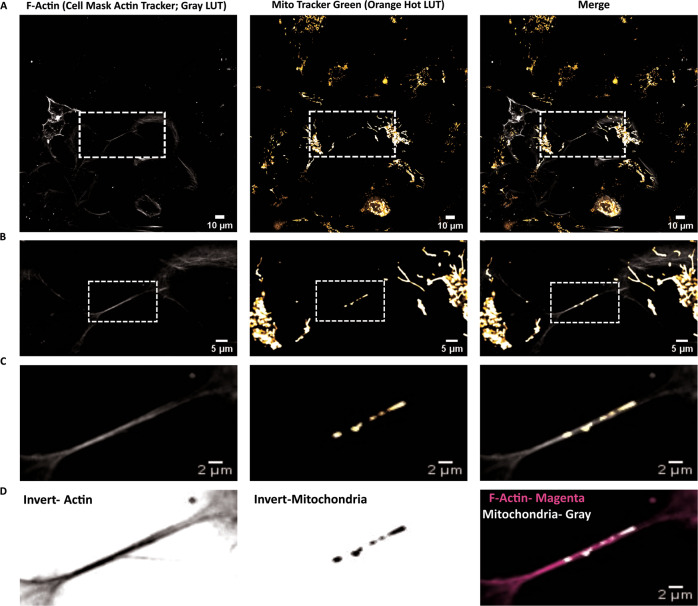
Functional TNTs are formed between microglia. **A** Single, middle stack image of microglial cells stained for F-Actin with cell mask deep red actin tracker (grey pseudo-coloured; left panels) and mitochondria with MitoTracker green(orange pseudo-coloured, middle panels). **B** Zoomed images from the ROIs boxed in **A**. **C**, **D** Zoomed images from the ROIs boxed in **B**. Image snapshots were acquired using Nikon Eclipse Ti2 spinning disk microscope. LUTs were created using FIJI.

Despite these morphological characteristics, an essential requirement for intercellular connections to be classified as TNTs is their ability to transfer cargoes between the connected cells. A major intracellular component commonly reported to be transferred via TNTs between different cell types and experimental conditions is mitochondria (reviewed in [22]). Towards this end, the presence of mitochondrial particles inside F-Actin containing TNTs connecting two microglia (Fig. 3A–D) supported the functional nature of these TNTs, which was further assessed by live imaging (see below, Supplementary movie 3).

### Functional TNTs are formed between neuronal cells and microglia

**Fig. 4:**
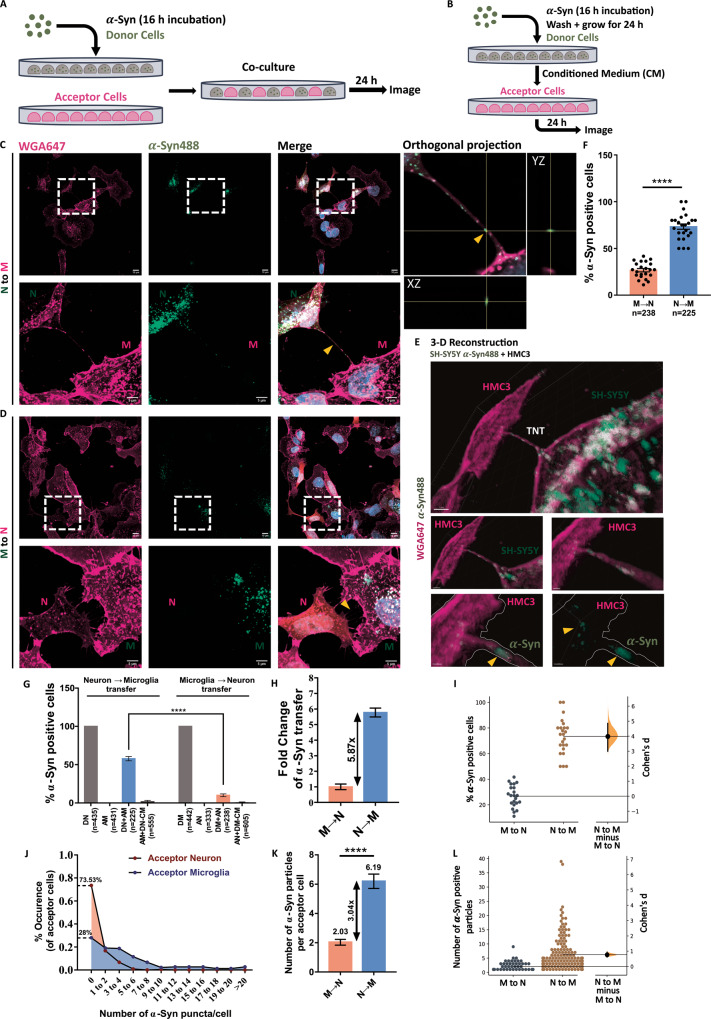
Movement of *α*-Syn from SH-SY5Y to HMC3 cells via TNTs. **A** Schematic representation of co-culture strategy used to assess for transfer. **B** Schematic representation of secretion control. **C** In a co-culture system, TNTs connect donor neuronal cells with acceptor microglia (N → M transfer). Lower panels are zoomed images of the ROI boxed in the upper panels. ‘N’ represents SH-SY5Y neuronal cells and ‘M’ represents HMC3 microglia. The yellow arrowhead in the orthogonal projection panel depicts *α*-Syn puncta inside TNT. **D** Co-culture of donor microglia with acceptor neuronal cells (M → N transfer) show the presence of TNTs between two cell types (lower panels), but without any *α*-Syn puncta within them (yellow arrowhead). **E** Imaris-based 3-D reconstruction of connected cells in **C**. **F** Proportion of acceptor cells positive for *α*-Syn puncta in a co-culture system. *N* = 3 independent experiments, *n* = 238 acceptor cells for M → N transfer and *n* = 225 acceptor cells for N → M transfer; Unpaired Student’s t-test; ****p < 0.0001. **G** Compiled representation of *α*-Syn transfers between both cell types, normalised for secretion control; One-Way ANOVA with Tukey’s *post-hoc*; ****p < 0.0001. **H** Fold-change difference between the two groups (M → N and N → M transfers) indicate a 5.87-times increase in the movement of *α*-Syn from neuronal cells to microglia. **I** Estimation statistics plot corresponding to **F**. **J** Distribution pattern of the number of *α*-Syn puncta transfer in acceptor cells. Higher value for “0” is indicative of more instances of no transfer. **K** Average number of *α*-Syn particles per acceptor cell. *N* = 3 independent experiments, *n* = 59 SH-SY5Y acceptor cells for M → N transfer and *n* = 160 HMC3 acceptor cells for N → M transfer; Unpaired Student’s t-test, ****p < 0.0001 **L**. Estimation statistics plot to correspond to **K**. Error bars represent mean ± SEM.

Although TNTs have been reported to form between neurons, and between microglia, in homotypic cultures, their presence between neurons and microglia has not been assessed. Towards this, in this study we used human neuroblastoma cell line SH-SY5Y, a well-established in vitro model to study TNTs under different (patho)physiological conditions [25, 33, 44]. Using a co-culture strategy between SH-SY5Y cells and HMC3 microglial cells (Fig. 4A), and normalising it to secretion-mediated transfer (Fig. 4B, described in materials and methods), we could observe TNTs connecting the two cell populations. To assess the functionality of these TNTs, we first loaded SH-SY5Y and HMC3 cells with *α*-Syn and measured the aggregate uptake ability of these cells at different time points (Fig. S1A, B). After 16 h of incubation with *α*-Syn, both SH-SY5Y and HMC3 cells had increased level of fibrils compared to other time points (Fig. S1C-D). However, SH-SY5Y neuronal cells had relatively higher abundance of fibrils compared to HMC3 microglial cells (Fig. S1E). Next, the *α*-Syn containing SH-SY5Y cells (donor cells) were co-cultured with HMC3 microglia naïve cells (acceptor cells) for 24 h before measuring the transfer of *α*-Syn aggregates to microglia by quantitative confocal microscopy (see materials and methods) (Fig. 4C, F, G, S2A, upper panels). We found that 57.85% of acceptor cells were positive for *α*-Syn puncta (N → M transfer) (Fig. 4F). However, when *α*-Syn-loaded microglia were the donor cells and naïve neuronal cells were acceptors (Fig. S2A lower panels) (M → N transfer), the extent of transfer was limited to 10.02%, lesser by 5.87 folds (Fig. 4D, F, G, H). Orthogonal projection (Fig. 4C) and 3-D reconstruction of the TNT connecting donor neuronal cells with acceptor microglia (Fig. 4E, supplementary movie 1) demonstrate the presence of *α*-Syn within the TNT. With a high value of Cohen’s d (Fig. 4I), the effect size of the difference was substantial. Additionally, the number of *α*-Syn puncta per acceptor cell was 3.04-fold higher in microglia as opposed to neuronal cells (Fig. 4J, K, L). 73.53% of acceptor neuronal cells did not receive any *α*-Syn from donor microglia, whereas only 28% of acceptor microglia were negative for *α*-Syn signal (Fig. 4J). Importantly, these values were all normalized with secretion control (see materials and methods) (Fig. 4B, S2B). The extent of secretion-based transfer was, however, only marginally increased (by 1.23-folds) from neuronal to microglial cells (Fig. S2E), supporting the relevance of cell-to-cell contact-mediated pathway for N → M aggregate transfer over secretion.

To directly demonstrate that TNTs were effectively transferring *α*-Syn aggregates between co-cultured cells we used live confocal microscopy. By this approach we could observe *α*-Syn puncta moving in TNTs from neuronal cells to microglia over a period of 30 minutes (Fig. S3A, B, supplementary movie 2). Overall, these data suggest that burdened neuronal cells actively relay *α*-Syn aggregates to microglia mainly using TNT-mediated transfer. The contrary, however, is not true, suggesting that microglia might play a neuroprotective role by accepting these aggregates, rather than contributing to aggregate spreading.

### Microglia provide mitochondria to neuronal cells

**Fig. 5:**
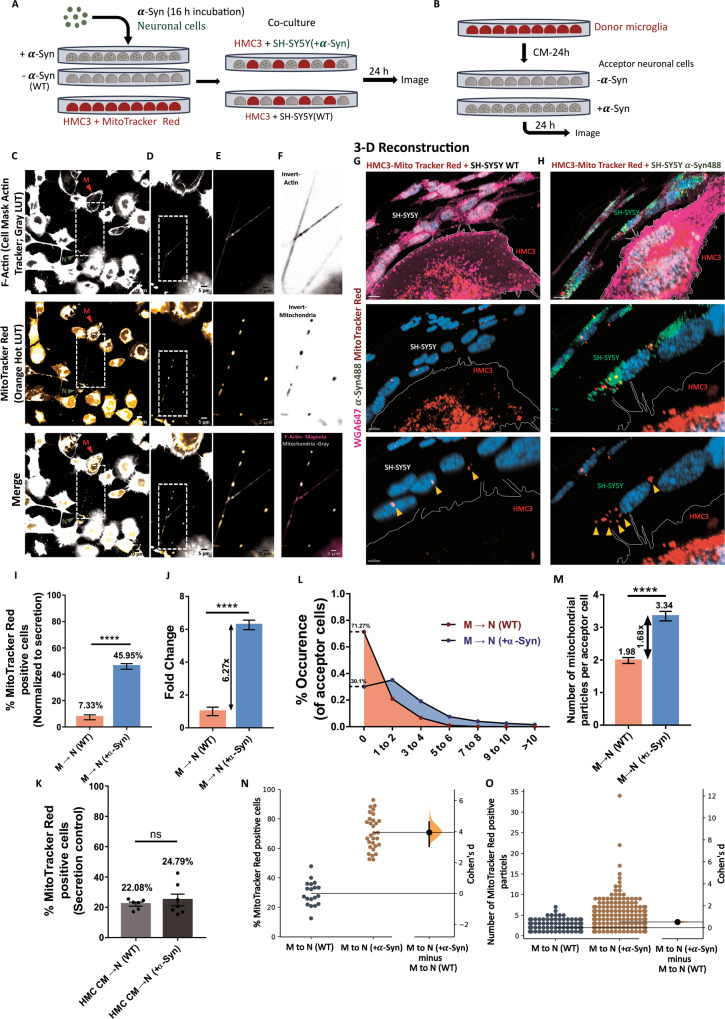
Movement of mitochondria from HMC3 to SH-SY5Y cells. **A** Schematic representation of the co-culture strategy used to assess transfer. **B** Schematic representation of secretion control. **C**–**F** Single, middle stack images of neuron-microglia co-culture stained for F-Actin with cell mask deep red actin tracker (grey pseudo-coloured, upper panels) and mitochondria with MitoTracker red (orange pseudo-coloured, middle panels). White, dashed boxes indicate the ROI for zoomed images. Images were acquired using Nikon Eclipse Ti2 spinning disk microscope (also see Fig. S4). LUTs were created using FIJI. ‘N’ represents SH-SY5Y neuronal cell, indicated with green arrowhead, and ‘M’ represents HMC3 microglia, indicated with red arrowhead. **G**, **H** Imaris-based 3-D reconstruction of microglia-derived mitochondrial particles in SH-SY5Y acceptor cells with *α*-Syn aggregates **H**, or without them **G**. Yellow arrowheads point towards mitochondrial particles inside SH-SY5Y cells. **I** Proportion of healthy- M → N (WT) and unhealthy- M → N ( + *α*-Syn) acceptor cells positive for mitochondrial particles. *N* = 3 independent experiments, *n* = 630 healthy (WT) acceptors and n = 638 unhealthy (+*α*-Syn) acceptors; unpaired Student’s t-test, ****p < 0.0001. **J** Fold change of difference between the two acceptor populations in receiving mitochondrial particles from microglia depict a 6.27-times increase for unhealthy neuronal acceptor population, Unpaired Student’s t-test ****p < 0.0001. **K** Proportion of healthy- M → N (WT) and unhealthy- M → N ( + *α*-Syn) acceptor cells positive for mitochondrial particles in secretion control. *N* = 3 independent experiments, *n* = 634 healthy (WT) acceptor cells and *n* = 605 unhealthy (+*α*-Syn) acceptor cells; Unpaired Student’s t-test; ns: non-significant. **L** Distribution pattern of the number of mitochondrial puncta transfer in acceptor cells. Higher value for “0” is indicative of more instances of no transfer. (**M**) Average number of mitochondrial particles in acceptor neuronal cells. *N* = 3 independent experiments, *n* = 181 for healthy (WT) acceptors and *n* = 446 for unhealthy (+*α*-Syn) acceptors. **N**, **O** Estimation statistics plots corresponding to un-normalised transfer in **I** and M respectively. Error bars represent mean ± SEM.

From our observation of a skewed directionality of *α*-Syn transfer, we next asked the possible functional significance of TNTs from a microglial perspective, and whether microglia could use TNTs to preferentially transfer materials to SH-SY5Y cells. An important pathological consequence of *α*-Syn accumulation in neuronal cells is mitochondrial damage. With the hypothesis of microglia providing neuroprotective functions to neuronal cells, we tested the possibility that microglial cells could supplement mitochondria to *α*-Syn-loaded SH-SY5Y cells. We co-cultured HMC3 cells labelled with MitoTracker Red CMXRos (donor cells) and SH-SY5Y with (+*α*-Syn) or without (-*α*-Syn; WT) fibrils (acceptor cells) (Fig. 5A). Microglial conditioned medium was added to both groups of acceptor neuronal cells to normalise for secretion control (Fig. 5B, described in materials and methods). As above, functional TNTs were observed between neuronal cells and microglia (grey connections, pseudo-coloured), containing mitochondria (orange particles, pseudo-coloured) within them (Fig. 5C–F, S4A). We observed a 6.27-fold increase in mitochondrial transfer to *α*-Syn loaded (45.95%) as compared to WT SH-SY5Y cells (7.33%) (Fig. 5I, J, N). On the other hand, secretion-mediated transfer was comparable for both groups (Fig. 5K). Besides the higher percentage of acceptor cells, we also observed a 1.68-folds increase in the number of mitochondrial particles received by *α*-Syn loaded neuronal cells compared to WT cells (Fig. 5G, H, L, M, O) (average 3.34 mitochondrial particles in *α*-Syn loaded cells compared to 1.98 particles in WT cells). Furthermore, only 30.1% of the *α*-Syn loaded cells did not receive any mitochondria, while 71.27% of WT cells were negative for any mitochondrial signals (Fig. 5L). Using time-lapse imaging, we could demonstrate the movement of mitochondria in TNTs from microglia to *α*-Syn-loaded neuronal cells (Fig. S4, Supplementary movie 3). Of interest, we could also observe co-transfer of *α*-Syn aggregates from neuronal cells to microglia, and mitochondria from microglia to neuronal cells along the same tube (Fig. S5, Supplementary Movie 4). Taken together, bi-directional transfer of *α*-Syn and mitochondria is indicative of the active distribution of materials between the connected cells, wherein unhealthy SH-SY5Y cells receive metabolic support from microglia, which in turn receive “toxic” aggregates.

### *α*-Syn aggregates increase homotypic TNT formation

**Fig. 6:**
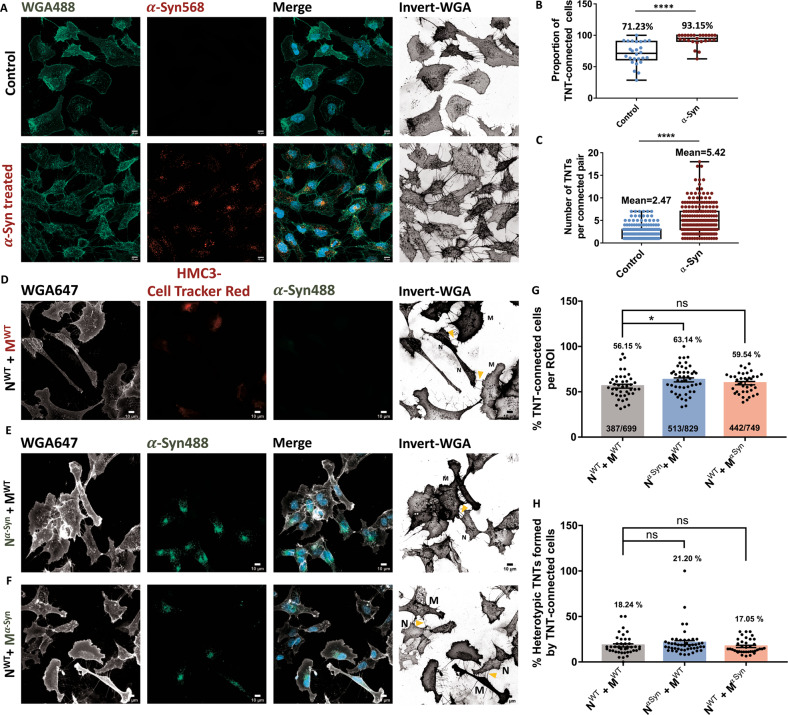
*α*-Syn exposure increases homotypic TNTs between HMC3 microglia, but not heterotypic TNTs between SH-SY5Y neuronal cells and HMC3 microglia. **A** Microglia stained for membrane (WGA, green) depicts increased global connectivity between cells in the presence of *α*-Syn (lower panels), as compared to the control group (upper panels). **B** Proportion of TNT-connected cells. *N* = 3 independent experiments, *n* = 252 cells for control group, *n* = 345 cells for *α*-Syn group; Unpaired Student’s t-test; ****p < 0.0001. **C** The average number of TNTs between connected pairs; *N* = 3 independent experiments, *n* = 172 connected pairs for control group, *n* = 200 connected pairs for *α*-Syn group; Unpaired Student’s t-test; ****p < 0.0001. **D** Co-culture of WT SH-SY5Y neuronal cells (N) with HMC3 microglia loaded with cell tracker red (M). Yellow arrowheads point towards heterotypic TNTs connecting the two cell types. **E** Co-culture of SH-SY5Y cells loaded with *α*-Syn (N) and WT HMC3 microglia (M). Yellow arrowheads point towards heterotypic TNTs. **F** Co-culture of WT SH-SY5Y cells N and HMC3 microglia loaded with *α*-Syn (M). Yellow arrowheads point towards heterotypic TNTs. **G** The proportion of TNT-connected cells in the field of view. *N* = 3 independent experiments, numbers of cells analysed per group mentioned within the respective bar graphs; One-Way ANOVA with Tukey’s *post-hoc*, ns: non-significant, *p < 0.05. **H** Proportion of TNTs in **G** that are heterotypic. *N* = 3 independent experiments, *n* = same as **G**; One-Way ANOVA with Tukey’s *post-hoc*, ns: non-significant. Error bars represent mean ± SEM.

Finally, we asked whether the presence of aggregates would affect TNT formation between SH-SY5Y neuronal cells and HMC3 microglia, as shown previously for CAD neuronal cells [27]. We, therefore, incubated both cell types with *α*-Syn for 16 h before assessing TNT formation. Compared to control conditions (Fig. 6A- top panel), we found a 21.92% increase in the proportion of TNT-connected microglia in the *α*-Syn treated group (Fig. 6A bottom panel, 6B). Besides the percentage of connected cells, an important question that arises is if the number of connections between cells also increase upon *α*-Syn exposure. We found an increase of more than 2-folds in the number of TNTs per connected pair when cells were treated with *α*-Syn (Fig. 6C), suggesting that microglia alter their morphological profile to form intercellular connections upon exposure to *α*-Syn. Similarly, SH-SY5Y cells increase intercellular connections by 23.42% in the presence of *α*-Syn (Fig. S6A, B). Then, to assess for the effect of *α*-Syn on heterotypic TNTs, one cell type loaded with *α*-Syn was co-cultured with the other cell type without *α*-Syn (N^α-Syn^ + M^WT^; and N^WT^ + M^α-Syn^; Fig. 6E, F), and compared with heterotypic TNTs formed in the absence of *α*-Syn (N^WT^ + M^WT^; Fig. 6D). Normalised to the percentage of connected cells (Fig. 6G, Eq. 1 in materials and methods), the number of heterotypic TNTs was not influenced by the presence of *α*-Syn (Fig. 6H). This, taken together with our previous results of *α*-Syn transfer analysis (Fig. 4), suggest that the elevated transfer of aggregates does not require a concomitant increase in heterotypic connections.

## Discussion

TNTs represent a direct mode of communication between cells of the same or different types. First reported to be formed between PC12 cells, subsequently several other cell types have been shown to form TNTs, supporting the potential involvement of these long-range membranous channels in health and diseases [20, 22, 23, 41]. TNTs have been implicated to play crucial roles early during development [45], as well as in neurodegenerative diseases, classically manifesting later in life [46]. TNT-mediated transfer of protein aggregates from an unhealthy to a naïve cell aid in the propagation of the pathology by allowing the seeding of aggregates in acceptor cells [28]. On a parallel tangent, TNTs can also facilitate the dilution of aggregates in a cell by sharing it with connected cells for eventual degradation. We have previously shown that neurons transfer *α*-Syn aggregates to astrocytes via TNTs where these fibrils were degraded [29]. Besides astrocytes, microglia are major, yet controversial, players in ND pathologies and their progression. On one hand, microglia can engulf and degrade *α*-Syn fibrils released by neurons to prevent neurodegeneration [19], while on the other hand, these cells can also directly (via release of matrix metalloproteases) and indirectly (via secretion of neurotoxic factors) influence death of neurons [6]. Of interest, an elegant study has shown that microglia form TNT-like connections with each other, enabling the distribution of *α*-Syn aggregate load between microglia and their subsequent degradation [32]. These data could represent a double-edged sword: if microglia were to transfer *α*-Syn aggregates to neurons, despite microglia’s ability to degrade aggregates, it would contribute to the spread of pathology. On the other hand, if there were a uni-directional transfer of aggregates from neurons to microglia, then these cells would perform a protective function. Thus, the question arises as to what the extent of TNT-mediated intercellular communication between neurons and microglia is, and the involvement of the latter in ND pathology prevention and/or spreading. Heterotypic TNTs have been show to facilitate transfer of aggregates between different cell types (such as neurons-astrocytes and neurons-pericytes) [29, 31]. However, no study has assessed the presence and role of TNTs between neurons and microglia.

Using HMC3 cell line, our quantitative data demonstrate that around 40% of microglia cells in culture are connected by TNTs under normal growing conditions, highlighting the potential importance of these structures (Fig. 1A, B). Most of the TNTs had lengths ranging from 10 to 20 μm, with several of them extending up to 40 μm (Fig. 1C, D). Despite previous reports indicating that cells of myeloid origin have thick TNTs [42, 47], we found that ~70% of these connections were thin, F-Actin containing TNTs, while only ~30% were thick, F-Actin and microtubule containing TNTs (Fig. 2A–E). Furthermore the extent of *α*-Tubulin presence was categorised to be “low”, “partial”, or “complete”. Only a very low proportion of TNTs had high presence of Tubulin. TNTs that contained Tubulin had higher diameter than those containing only F-Actin, in accordance with our current understanding of “thick” and “thin” TNTs [41] (Fig. 2F). However, diameter measurement of TNTs is only approximate with fluorescence microscopy. Ultrastructure analysis with electron microscopy techniques will better represent the near-actual diameter of these connections [44]. The predominant presence of F-Actin is validated by previous data from Scheiblich and colleagues showing that knocking out ROCK2, a regulator of Actin polymerisation, significantly increases the exchange of *α*-Syn aggregates between microglia [32]. On the other hand, it will be interesting to relate the cytoskeletal composition with the formation of TNTs, as to whether the presence of tubulin polymers indicates a maturation of these structures, and/or with the transfer abilities of TNTs.

Here we show that microglial TNTs are functional as they contain mitochondrial particles and support their transfer between cells (Fig. 3A–D, supplementary movie 3). These data support recent findings showing transfer of *α*-Syn aggregates in TNTs between microglia [32]. To understand whether microglia are involved in the spreading of aggregates to neuronal cells or exert a protective function by up-taking *α*-Syn aggregates from these cells, we carried quantitative and qualitative microscopy studies in co-culture of the two cell types. We demonstrate that functional TNTs form between HMC3 microglia and human neuronal SH-SY5Y cells. Quantitative assessment of the transfer of *α*-Syn aggregates show that the extent of aggregate transfer is quite low from microglia to neuronal cells (Fig. 4F, G, H), while we observe a 5.87-folds increase in the transfer from neuronal to microglial cells (Fig. 4H). The effective bias of aggregate transfer is strengthened by our observation that SH-SY5Y neuronal cells have a relatively high abundance of aggregates (2.18-folds compared to HMC3 microglia) within their small volume and would therefore require to employ a mechanism of dissipating the load (Fig. S1E). Although SH-SY5Y cells had only a 2.18-folds increase in *α*-Syn level, the 5.87-folds increase in transfer indicates an active effort by these neuronal cells towards this. Given a major function of microglia is phagocytosis and degradation of pathogenic materials, we speculate that this could be a way for neuronal cells to lower their aggregate burden, suggesting that microglia could exert a protective function for neurons, similar to what we had previously proposed for astrocytes [29], rather than contributing to the spreading of the pathology. This hypothesis is supported by the recent finding that acceptor microglia are involved in the active degradation of *α*-Syn aggregates after receiving it from donor cells [32]. Furthermore, we confirm and extend previous findings showing that microglia increase intercellular connectivity in the presence of *α*-Syn aggregates. Not only is the global connectivity increased (Fig. 6A- lower panels, B), but the number of TNTs connecting two cells are also increased by 2.19-folds (Fig. 6C). SH-SY5Y neuronal cells also increase TNT connections between them in the presence of aggregates (Fig. S6A, B), similar to what has been observed previously for CAD neuronal cells [27]. However, the number of heterotypic connections between two cells are not affected by the presence of *α*-Syn (Fig. 6H), suggestive of a mechanism of aggregate transfer wherein cells utilise the pre-existing connections with an increased rare of transfer. This could also suggest differential regulation of homotypic and heterotypic TNT formations, with the former being sensitive to aggregate presence, a hypothesis that remains to be tested. In the presence of *α*-Syn aggregates, the molecular landscape of microglia changes from a homoeostatic to a reactive state, with upregulation of pro-inflammatory genes, including chemotactic genes [32]. We speculate that such drive of microglia to a pro-inflammatory state might potentially alter the cellular cytoskeleton dynamics of these cells, allowing them to form more intercellular connections with each other, similar to what has been shown for HIV-1 infected macrophages [48] and dendritic cells [49]. The (patho)physiological advantage of such connections is an exchange of aggregates for eventual clearance. *α*-Syn-mediated increase in connections could therefore be a dose-dependent (concentration of α-Syn) or a time-dependent (period of incubation) process, both of which need to be further elucidated.

Next, we asked the question of whether microglia would use TNTs as donor cell to supply material to healthy or unhealthy neuronal cells. We observed a significant increase (6.27-folds) in the transfer of mitochondrial particles from microglia to “unhealthy” SH-SY5Y loaded with *α*-syn fibrils, as opposed to “healthy” ones (not-loaded with aggregates) (Fig. 5G, H, I, J). Interestingly in PC12 neuronal cells, the transfer of mitochondria from a healthy cell to an unhealthy, UV-irradiated cell has been reported to rescue the phenotype of the latter [50]. Additionally, transfer of functional mitochondria from healthy pericytes to oxygen and glucose-deprived (ischaemic) astrocytes via TNTs rescue them from apoptosis [51]. Thus, we speculate that this could be a possible way of rescuing metabolic health of *α*-Syn burdened cells. Interestingly, we observed simultaneous, bi-directional transfer of *α*-Syn and mitochondria (Supplementary movie 4), suggestive of active interactions between the two cells working in concert to restore homoeostatic balance. Ultrastructural analysis of neuronal TNTs using correlative FIB-SEM has identified bundles of individual TNTs (iTNTs) that connect the opposing cells and are held together by N-Cadherin. Such iTNTs could be aligned in parallel or anti-parallel fashions [44]. Consequently, our observation of bi-directional cargo movement can be facilitated by iTNTs that maintain opposing polarities, thereby allowing uni-directional material transfer in both directions. Alternatively, this could be mediated by a single, thick TNT containing both actin and microtubules.

In conclusion, our work demonstrates for the first time the presence of functional TNTs between neuronal and microglial cell lines, that allow for material transfer in a selective manner. Protein aggregates are transferred with a high degree of bias from neuronal cells to microglia, whereas mitochondria is transferred preferably in the opposite direction, thereby supporting the idea of protective roles of microglia in NDs. This paves way for further investigation using murine and human primary cells, which would give subsequent insights into the mechanisms of neurodegenerative pathology spreading, especially on the involvement of microglia in NDs.

## Supplementary information

Supplementary material

Figure S1

Figure S2

Figure S3

Figure S4

Figure S5

Figure S6

Supplementary movie 1

Supplementary movie 2

Supplementary movie 3

Supplementary movie 4

Reproducibility Checklist

## Data Availability

All data related to the manuscript are available in the main, or supplementary figures. Data can be made available upon request.
