# Supplementary material for: Tunnelling nanotubes between neuronal and microglial cells allow bi-directional transfer of α-Synuclein and mitochondria
Source: Cell Death Dis. 2023 May 18;14(5):329. doi: 10.1038/s41419-023-05835-8 (PMC10195781; doi:10.1038/s41419-023-05835-8)
Supplement: Supplementary file 1 — Supplementary material [file 41419_2023_5835_MOESM1_ESM.docx]

**Supplementary Information**

**Title: Tunneling nanotubes between neuronal and microglial cells allow bi-directional transfer of** $\boldsymbol{\alpha}$**-Synuclein and mitochondria**

**Running title: Neuron-microglia communication through tunneling nanotubes**

Ranabir Chakraborty^1,2^, Takashi Nonaka^3^, Masato Hasegawa^3^, Chiara Zurzolo^1,4,*^

^1^Institut Pasteur, Université Paris Cité, CNRS UMR 3691, Membrane Traffic and Pathogenesis, F-75015, Paris, France

^2^Université Paris Saclay, Gif-sur-Yvette, Paris 91190, France

^3^Dementia Research Project, Tokyo Metropolitan Institute of Medical Science, Tokyo, Japan

^4^Department of Molecular Medicine and Medical Biotechnology, University of Naples Federico II, Naples, Italy.

*Correspondence: [chiara.zurzolo@pasteur.fr](mailto:chiara.zurzolo@pasteur.fr)

**Supplementary figure legends**

**Fig. S1: Loading of α-Syn in SH-SY5Y neuronal and HMC3 microglial cells.** (A-B) SH-SY5Y cells (A) and HMC3 cells (B) incubated with α-Syn fibrils for different time points as indicated. (C-D) Intensity per unit area of $\alpha$-Syn in SH-SY5Y (C) and HMC3 (D) cells; N=3 independent experiments, n=125 cells per group; One-Way ANOVA with Tukey’s *post-hoc*; ns: non-significant, *p<0.05, ****p<0.0001. (E) Compiled intensity per unit area of $\alpha$-Syn fibrils in the two cell types. N=3 independent experiments, n=125 cells per group; Two-Way ANOVA with Tukey’s *post-hoc*; ns: non-significant, ****p<0.0001. Error bars represent mean ± S.E.M. Related to figure 4.

**Fig. S2:** $\boldsymbol{\alpha}$**-Syn transfer assay between SH-SY5Y and HMC3.** (A) Mono-cultures of donor neuronal cells and acceptor microglia (upper panels, N→M transfer), and donor microglia and acceptor neuronal cells (lower panels, M→N transfer). (B) Images of acceptor cells (microglia in upper panels, neuronal cells in lower panels) to assess for secretion-based transfer. (C-D) Distribution pattern of the number of $\alpha$-Syn puncta in acceptor microglia (C) and acceptor neuronal cells (D) for secretion control experiments. (E) Average number of $\alpha$-Syn particles received by acceptor neuronal and microglial cells via secretion. N=3 independent experiments, n=605 acceptor neuronal cells and n=555 acceptor microglial cells; Unpaired Student’s t-test; *p<0.05. Error bars represent mean ± S.E.M. Related to Fig. 4.

**Fig. S3:** (A-B) Time-stamps at every 5 minutes interval of $\alpha$-Syn transfer from neuronal (green $\alpha$-Syn loaded) to microglial cells. Related to Fig. 4, and supplementary movie 2.

**Fig. S4:** (A) Time-stamps at every 5 minutes interval of mitochondrial transfer from microglia (M) to $\alpha$-Syn loaded neuronal cells (N). Yellow and gray arrowheads point towards mitochondrial particles inside TNTs. Related to Fig. 5, and supplementary movie 3.

**Fig. S5:** (A) Time-stamps at every 5 minutes interval of bidirectional $\alpha$-Syn transfer from neurons (green) to microglia (red) and mitochondria from microglia (red) to neurons (green). Upper arrowhead points towards $\alpha$-Syn aggregate, and the lower arrowhead points towards mitochondrial particle. Related to Fig. 4 and 5, and supplementary movie 4.

**Fig. S6:** $\boldsymbol{\alpha}$**-Syn exposure increases homotypic TNTs between SH-SY5Y neuronal cells.** (A) SH-SY5Y cells stained for membrane (WGA, green) increase inter-cellular connectivity (yellow arrowheads) in the presence of $\alpha$-Syn (lower panels), as compared to the control group (upper panels). (B) Proportion of TNT-connected cells. N=3 independent experiments, n=491 cells for control group and 552 cells for $\alpha$-Syn treated group; Unpaired Student’s t-test; ****p<0.0001. Related to Fig. 6.

**Supplementary movie legends**

**Supplementary movie 1:** 3-D reconstruction (Imaris) of $\alpha$-Syn in TNT connecting donor neuronal cells with acceptor microglia. SH-SY5Y neuronal cells loaded with $\alpha$-Syn co-cultured with HMC3 microglia. Related to Fig. 4C, E.

**Supplementary movie 2:** Movement of $\alpha$-Syn from neuronal cell to microglia (yellow arrowhead). Respective cells were co-cultured for 24 h, followed by time-lapse imaging using Nikon Ti2 spinning disk, 60X/1.4 NA oil objective. Images were acquired every minute for 30 minutes (30 frames) in green ($\alpha$-Syn488) and far-red (cell mask deep red actin tracker) channels, and a video created using FIJI. Related to Fig. 4.

**Supplementary movie 3:** Movement of mitochondria from microglia to $\alpha$-Syn loaded neuronal cell (yellow and gray arrowheads). Respective cells were co-cultured for 24 h, followed by time-lapse imaging using Nikon Ti2 spinning disk, 60X/1.4 NA oil objective. Images were acquired every minute for 30 minutes (30 frames) in green ($\alpha$-Syn488), red (MitoTracker Red CMXRos), and far-red (cell mask deep red actin tracker) channels, and a video created using FIJI. Far-red channel represented in Fig. 5C-F, and not in the video for the purposes of better clarity. Related to Fig. 5.

**Supplementary movie 4:** Bi-directional movement of $\alpha$-Syn from neuronal cell to microglia and mitochondria in the opposite direction (green and yellow arrowheads for distinct $\alpha$-Syn particles; gray arrowhead for mitochondrial particle). Respective cells were co-cultured for 24 h, followed by time-lapse imaging using Nikon Ti2 spinning disk, 60X/1.4 NA oil objective. Images were acquired every minute for 30 minutes (30 frames) in green ($\alpha$-Syn488) and red (MitoTracker Red CMXRos) channels, and a video created using FIJI. Related to Figs. 4 and 5.
